# From Our Correspondents

**Published:** 1984-04

**Authors:** 


### From Our Correspondents

**Frenchay on Television**

By some unusual coincidence Frenchay Hospital in one form or another featured twice in the course of one week on the National Television System recently. Whether this means that we have mass media appeal or that we are known by the programme writers as an easy touch is not clear, but the occurrence surely merits a comment in the annals of local hospital events.

The week got off to a rousing start with a 'Your Life in Their Hands' programme straight from the Department of Thoracic Surgery. This seemed appropriate, as, historically, Frenchay Hospital was a Chest Hospital before being taken over by the National Health Service in 1948. The two stars on the programme were Mr. K. Jeyasingham, with Dr. P. Simpson anaesthetising, and I quote from their report:

'Our recent experience at Frenchay ("Your Life in Their Hands", BBC 28/2/84) televising surgical treatment of a patient with a sinister shadow in the chest X-ray was far less daunting than we had anticipated. Together with the Producer, we had decided that the programme would show that the medical staff should be both professional and human, and that irrespective of any sensational element the patient himself and his treatment should be of paramount importance. This was further enhanced by the Producer's desire to allow the audience to identify with the particular individual's surgical treatment.

'Although both of us had met and talked with the patient at length, the actual filming, both pre and intra-operatively, was totally unscripted and obviously did not allow repeated "takes" of any significance. Despite this, the lack of need for any enhanced lighting and the complete mobility and adaptability of the film crew allowed us to operate in a relatively normal, if somewhat slow fashion. The theatre did not need to be converted into a film studio, neither did we need to be made into film stars! There are some who feel that televising treatment of a patient is unethical and we think they are wrong, and we think that the final result was neither of these. We were not involved with the editing of the material and perhaps would have prefered the exclusion or omission of certain parts, but in general welcomed the experience and felt that what resulted was educational and informative.'

Later in the week, both the B.B.C. and I.T.V. descended in strength on the hospital once again to investigate the British Telecom Image Transfer System which was later to be broadcast on both the local and national network. This development arose from a demand for the transmission of images, initially thought of in terms of C.T. of intracranial lesions, from one hospital to another so that transfer of patients could be properly selected and controlled.

A recent meeting at the hospital involving representatives from four main C.T. manufacturers and several other electronic firms produced a discussion on different systems of image transfer, all apparently very expensive and in many cases impractical. The representative from British Telecom however gave a fascinating talk and display of a system that they themselves had evolved using the ordinary telephone wires which seemed to answer almost all the requirements, both in terms of image quality and expense. Dr. Ian Mackintosh, the senior physicist at Frenchay Hospital responsible for organising the meeting and stimulating interest writes about the system in the following way:

'For several years both the Neurosurgery and Neuroradiology Departments at Frenchay Hospital have shown considerable interest in the concept of moving images between X-ray scanning equipment in different hospitals and, in the broader context, from hospital to consulting room or even a doctor's home. Until recently the cost of such image transfer equipment and its interfacing to X-ray scanners has proved so complex that no cost effective solution has been produced.

'Quite independently, British Telecom's Central Research Laboratories at Martlesham have been developing an image transfer system for use in security surveillance areas. The system sends images through standard telephone wires at a resolution of 256×256 pixels each with a grey scale of 64 levels. The image transfer equipment is small enough to fit inside a brief case, and the final image can be displayed on a domestic television set if required. The images take a maximum of 30 seconds to transmit, and the receiving station has the capacity to store up to four images within its local memory for display and review purposes. A demonstration of the system transmitting CT Scanner images was given at Frenchay, and the reaction of clinicians to both the image quality and ease of use of the system has been very favourable.

'In operation, it will be expected that a patient would initially have investigations within the receiving hospital. If the opinion of a senior consultant were required, then a telephone call could be made to another hospital or even the consultant's home to discuss the case and, if required, through the same telephone connection, to transmit relevant X-ray and scan data. The viewing station can be plugged in at any site where the now standard British Telecom jack plug connection is used for the phone, and in this way the system can be moved from one location to another.

'A 3 month evaluation of the potential of the system has now been started, during which time the equipment will be used to transmit images between Frenchay, the B.R.I. and Gloucester, as well as to individual consultants' homes. By the end of the trial it is hoped that the full potential and areas of application of the system, including its wider use in linking G.P.s' surgeries to hospitals, will be considered.'

This system of image transfer may soon be in use in the hospitals in the Bristol area, so that first hand knowledge may soon be available. You have been warned!

J.L.G.T.

**On Appointment Committees**

The Ancient Romans revelled in gladiatorial contests and one-sided conflict between ravening beast and hapless victim. Their legacy remains with us—even in Medicine. The only differences are that we call them Appointments Committees and hold them in oak-panelled hospital board rooms. Those of us who masochistically decided to seek our long-term future within the hospital service are only too familiar with these 'trials by ordeal' and I just cannot fathom why we have put up with it for so long. I can remember with dread one occasion on which, at the end of a fraught interview, I could not find the door set into the wainscoting which would allow me to escape! On another a senior member of my profession 'took me apart' on a trifling point because he wished another candidate to obtain the post.

Now that it is my turn to sit in judgement, so to speak, I find the whole system even more inhumane than when I was the candidate, particularly for consultant appointments. The impressions of the moment, most of which are probably totally erroneous, carry far too much weight in the final analysis. The tendency for like to choose like whatever the candidate's qualifications is strong. The supplicant who has been totally committed clinically because his consultant has given him no alternative 'hasn't published enough'. Those who have striven against all odds to obtain an M.D. or M.Chir. and several publications are 'far too academic'. Often the one who gets the job is the nice, safe candidate who is going to be easy to get on with and who is not going to rock the boat.

What makes matters even worse is that in my day most competent clinicians could be fairly sure they would achieve what they wanted within a reasonable time. If they did not, there were escape routes which included general practice early in their career or emigration later. These options have virtually disappeared today and in addition there are many more candidates for each post than ever there were. Thus our 'ad hoc' tradition of apprentice-type training is failing our young colleagues badly, both in the manner in which it prepares them for their long-term future and in the way in which it gives them neither security for the present nor confidence for the future.

G.M.S.

**Prognosis**

*Some Personal Case Histories*

(1) Last week in my Out-Patient Clinic a sprightly eighty-three year old man walked in with a bronchial infection. During the course of the interview he told me that he was discharged from the Army in the First World War with V.D.H. and told he only had a year to live. I was unable to find any fault in the valves: he had a nervous tachycardia. Needless to say, his faith in the wisdom of our profession was rather limited!

(2) At the M.B. London finals in 1920 a clinical case was presented of a traumatic arterio-venous aneurysm in the neck. The candidate made the correct diagnosis. The examining surgeon drew the candidate aside and said 'I'm afraid he will only live a year or so'. At the M.B. London finals in 1950 the same patient was presented to the earlier candidate's daughter. She once more made the correct diagnosis. This time the examining surgeon said 'I don't know why he has lived so long!'

(3) A friend of ours who had previously had peritonitis was told after innumerable tests and ten years of infertility that there was no chance of a pregnancy. She therefore adopted two children and had a house designed and built for a two child family, but within a year conceived a child of her own!

(4) A surgical colleague of ours in London made a confident diagnosis of inoperable bladder carcinoma at laparotomy. He gave a gloomy prognosis, and the patient sold up all his business interests in order to enjoy the last few months of his life. Some years later, in good health, he sued the surgeon for giving him such a false prognosis!

(5) A patient of mine aged seventy-five reported that twenty years before, in another town, she had been transferred to a home for the dying following a laparotomy at which 'widespread cancer' was found. She wisely only stayed in the home for a few weeks and discharged herself. Our investigations revealed polycystic disease of the liver and kidneys from which she eventually died in uraemia.

These somewhat extreme examples made me contemplate about our ability to give a reasonable prognosis. Honest practitioners in every branch of our profession must be able to quote similar stories. We are all shattered from time to time by the occasional fatal pulmonary embolus after a minor operation, or the fatal heart attack in the bran-eating keep-fit fanatic, but I venture to suggest that we can do more harm to more people by being excessively gloomy. It is so easy to fuel a cardiac neurosis by excessive restrictions on a patient's life, or to foster loss of function and independence after a stroke by relatives being over-protective. We can so easily encourage compensation neurosis by over-treatment and keeping patients off work too long. Many patients are signed off work for two weeks for a condition such as a respiratory infection when the doctor might only take two days off for the same complaint! Epileptic patients are almost literally wrapped in cotton-wool as relatives are terrified of being publicly castigated by a coroner if they have a fit whilst they are not watching over them.

I have a great respect for many of my self-employed patients such as farmers, bookies and shopkeepers. They have a determination to overcome their disabilities and return to work which often involves ignoring 'doctors orders'. They encourage me to be more tolerant, less restrictive and less dogmatic in my advice. Of course, we know the bad outlook for most people with malignant hypertension, secondary carcinoma, uraemia and so forth, but experience teaches us that some patients seem to tolerate their diseases or conquer them, thus refuting their doctor's gloom. For example, I had one obese patient who lived with malignant hypertension for twenty years; an adult patient who survived fifteen years with proven acute myeloid leukaemia, and two women who lived with uraemia for twenty years undialysed. I would put in a plea for us being as factual as we can concerning prognosis, whilst retaining some chink of optimism for patient and relatives alike.

H.G.M.

**Catting:[1] a new potential Health Hazard**

Once upon a time the columns in this *Journal* were respectably confined to 'professional' happenings. Dear reader, your Editor has given a commendably free brief to his most junior Correspondent. Alas, he—but hopefully not your Editor—has a current obsession with Cats. Let me explain why, and recapitulate. Two years ago on a 'Cater-for-Yourselves' farmhouse holiday in Wales our family acquired a ginger female kitten. A little quickly resulted,[2] but soon only a single offspring was left after the spectacular demise[3] of her Dam.

Now let me tell of further developments which threaten emotional tranquility, and even physical health in our household. In earlier days readers of the Bristol Medico-Chirurgical Journal would certainly have affected to be shocked by our family's rapid moral decline. Why, you ask? First, there was the problem of premature pubescent love. This was soon displayed in full degree by our second-generation, if furry, family immigrant. Half the feline neighbourhood rejoiced. Their male arpeggios frome fence tops rudely interrupted early morning slumbers in nearby conjugal bedrooms. Artfully, such communications were conducted well outside the range of water jugs, or even of old boots. The biological results were equally beyond any possible human interference.

Secondly, not long afterwards, quite unexpected results of these social encounters followed. By now our emancipated cat received—in recognition of her singular virtues and increasing independence—a metal collar tag with her home telephone number. Hitherto, not surprisingly with four teenagers, almost all incoming telephone calls to the house had concerned our own offsprings' love affairs: incipient, actual or dying. No one over the age of 18 even thought to answer the telephone.

Within days all that was changed. Weary and increasingly debilitated parents had to cope with a continuous battery of calls. '. . . I hope you don't mind . . . but I am ringing on behalf of your cat.' My cat? Our cat? Your cat?[4] With subsequent visits we have thus recently met poets, helpful medical students, trainee nannies, schoolgirls, women's libbers, waifs, strays, classical guitarists and even off-duty policemen on the cat's account. All callers have been charming, even if expensive on coffee, chat and lost sleep.

Gentle reader, please take a simple message to heart. Do hesitate before going on a holiday to rural Wales with children. Above all, have sense. Don't bring home to town any innocent country cat. It could prove a potential parental Health Hazard. Another, less flippant, warning about perils inherent in the warm-blooded pets of Bristol medical circles,[5] leads one to suggest that your families may be better served by a tortoise: at least they seem more discrete at bedtime.

Even so, it's probably best not to give their tortoise friends your telephone number. Who knows to what this column might otherwise degenerate in future?

### REFERENCES

1. Admittedly an unpleasing word, not perhaps felicitously, described as meaning "caterwauling, or, going after the opposite sex". Murray, J. A. H. et al. *Oxford English Dictionary*, vol. 2, 1933, p. 189; Partridge, E. *A Dictionary of Slang and Unconventional English*, Routledge and Kegan Paul, 5th Edition, 1961, p. 135. Possibly, hitherto justifiably obsolete since the 18th century.
2. Davies, J. D. (1984) Who cares about names? *Bristol Med.-Chi. J. 99*, in press.
3. Davies, A. R. (1983) Cat was killed by four dogs. *Bristol Evening Post* No. 15624, p. 48.
4. Such rhetorically progressive usage of possessive pronouns seems peculiar to certain strained domestic circumstances. More usually applied to human offspring. (*Personal experience(s)*).
5. Burton, J. L. (1983) Prolonged haemorrhage following nail clipping. *Lancet ii* 1484–1485.

